# Natural biomimetic nano-system for drug delivery in the treatment of rheumatoid arthritis: a literature review of the last 5 years

**DOI:** 10.3389/fmed.2024.1385123

**Published:** 2024-05-09

**Authors:** Jingyuan Li, Wei Li, Liping Zhuang

**Affiliations:** Beidahuang Group Mudanjiang Hospital, Mudanjiang, Heilongjiang, China

**Keywords:** rheumatoid arthritis, natural biomimetic, nano-system, drug delivery, treatment

## Abstract

Rheumatoid arthritis (RA) is a chronic systemic autoimmune disease characterized primarily by synovitis, leading to the destruction of articular cartilage and bone and ultimately resulting in joint deformity, loss of function, and a significant impact on patients’ quality of life. Currently, a combination of anti-rheumatic drugs, hormonal drugs, and biologics is used to mitigate disease progression. However, conventional drug therapy has limited bioavailability, and long-term use often leads to drug resistance and toxic side effects. Therefore, exploring new therapeutic approaches for RA is of great clinical importance. Nanodrug delivery systems offer promising solutions to overcome the limitations of conventional drugs. Among them, liposomes, the first nanodrug delivery system to be approved for clinical application and still widely studied, demonstrate the ability to enhance therapeutic efficacy with fewer adverse effects through passive or active targeting mechanisms. In this review, we provide a review of the research progress on the targeting mechanisms of various natural biomimetic nano-delivery systems in RA therapy. Additionally, we predict the development trends and application prospects of these systems, offering new directions for precision treatment of RA.

## 1 Introduction

Rheumatoid arthritis (RA) is an autoimmune disease characterized by bilateral inflammation of multiple joints. It involves the infiltration of synovial inflammatory cells in local joint cavities, leading to tenosynovitis, cartilage destruction, and bone erosion (1). In addition to the progression of joint inflammation and cartilage destruction, the extra-articular system is frequently affected during the course of the disease (2). Conventional drugs including nonsteroidal anti-inflammatory drugs (NSAIDs), disease-modifying anti-rheumatic drugs (DMARDs), glucocorticoids (GCs), and biologics are currently used for RA treatment (3, 4). These medications predominantly aim to suppress the immune response or the inhibit specific inflammatory mediators in order to alleviate symptoms associated with RA. However, the efficacy of drug treatment limited by the drug’s short effective half-life and its insufficient ability to specifically target diseased tissues, leading to poor clinical outcomes (5). Furthermore, upon *in vivo* administration, the drug is disseminated throughout the body, resulting in an elevated risk of side effects on extra-articular organs (6).

Nano-delivery systems emerge as a promising therapeutic strategy to enhance the efficacy of drugs and optimize their therapeutic outcomes. Clinical studies reported that nano-delivery systems have made remarkable contributions to the treatment of diverse diseases (7–9). Nano-delivery systems enhance drug solubility, prolong drug circulation time, reduce drug clearance, and deliver drugs to disease sites in a controlled manner (10, 11). In recent years, the design of multifunctional nanocarriers with sophisticated targeted drug delivery capabilities or transformable properties has gained significant attention (12, 13). These advancements enable smart drug delivery and aim to enhance therapeutic efficacy for RA. In brief, the integration of an efficient drug with a nano-delivery system holds promising potential as a therapeutic approach. Importantly, in comparison to exogenous nano-delivery systems (14, 15), the utilization of natural biomimetic nano-system for drug delivery offers superior biocompatibility, reduced cytotoxicity, and non-immunogenicity (16, 17). In this review, we focus on the utilization of natural biomimetic nanomaterials in the field of drug delivery for RA treatment, and discuss their advantages and limitations.

## 2 Methods

An extensive literature review was undertaken to understand the natural biomimetic nano-systems for drug delivery in the treatment of RA. A literature search was conducted on ScienceDirect, PubMed, and Web of Science for literature published between 2019 and 2023, using the keywords natural biomimetic, drug, nucleic acid, RNA, delivery system, endogenous albumin, extracellular vesicle, cell membrane, genetically engineered membrane, viral vectors, non-viral vectors, and nanoparticles (NPs) combined with rheumatoid arthritis. Other available resources were also used to identify relevant articles.

## 3 Molecular mechanisms of RA pathogenesis

**Figure 1:**
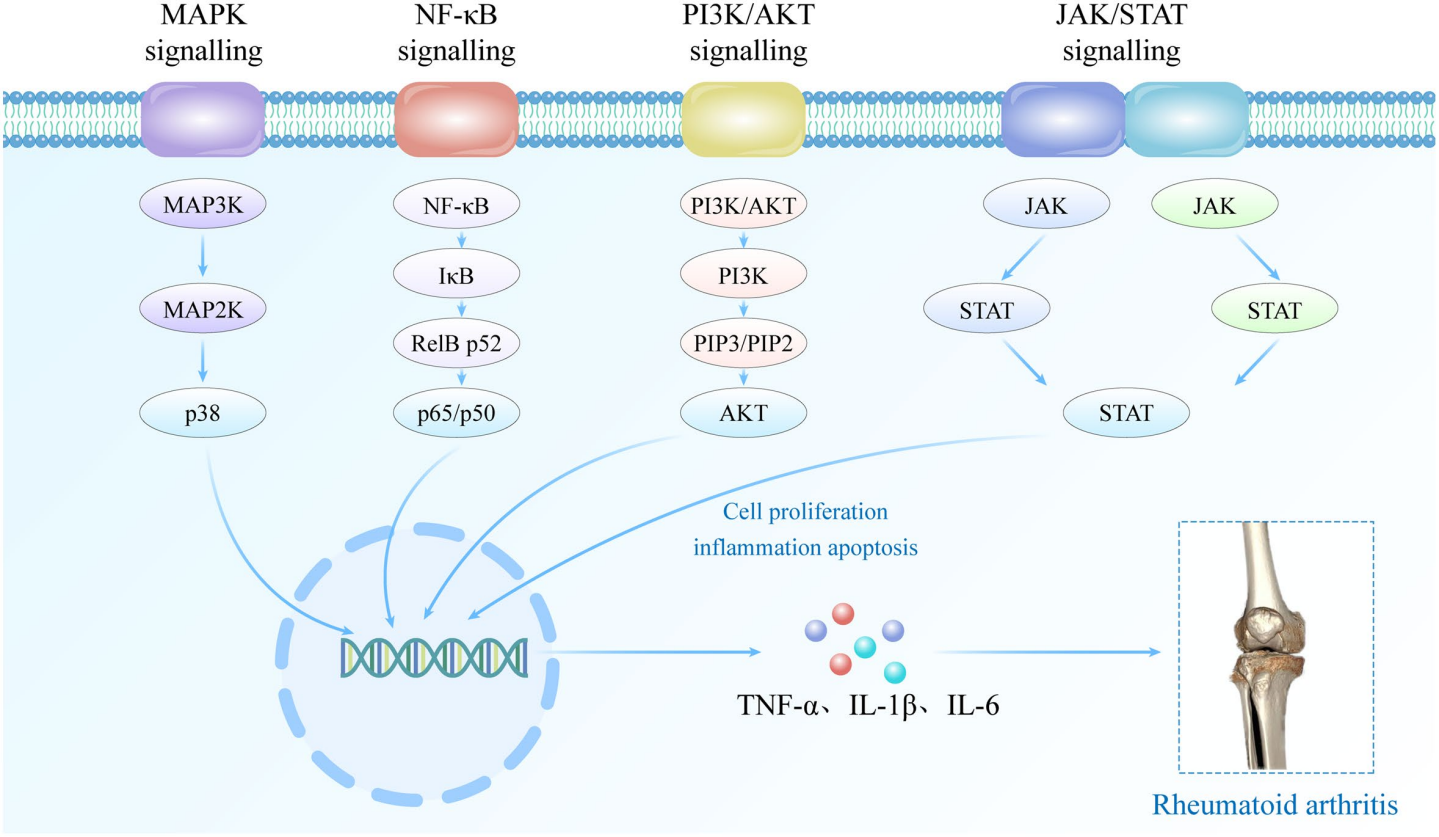
The role of signaling pathways in RA.

The occurrence and progression of RA are associated with dysregulated signaling pathways and autoimmune dysfunction. Abnormal regulation of signaling pathways (Figure 1), including MAPK, NF-κB, PI3K/AKT, JAK/STAT, among others, leads to abnormal expression of inflammatory cells and mediators such as fibroblast-like synoviocytes (FLSs), synovial macrophages, and other inflammatory mediators within the affected joint cavities (18). The interplay between multiple inflammatory cells and cytokines contributes to the inflammatory response in RA, leading to hyperactive immune system activity that drives the development and perpetuation of the disease (18, 19). In the early stages of RA pathogenesis, B cells secrete pro-inflammatory cytokines such as rheumatoid factor and anti-citrullinated protein antibody, which play a pivotal role in mediating T cell and macrophage activation (20). Upon activation, T cells and macrophages secrete inflammatory mediators such as tumor necrosis factor-α (TNF-α), matrix metalloproteinases (MMPs), interleukin-1 (IL-1) and IL-17. These mediators further exacerbate the inflammatory response, promote the formation of vascular opacities, and contribute to the damage of articular cartilage. In addition, immune cells such as dendritic cells and FLSs play a crucial role in mediating the pathophysiologic process of RA. In the advanced stages of immune system dysregulation during RA pathogenesis, these synovial cells undergo excessive proliferation and differentiate into tissue-invasive effector cells, thereby stimulating the formation of osteoclasts, ultimately resulting in progressive joint damage and the persistent presence of invasive inflammation within the synovial tissue (21).

## 4 Drugs delivery system

**Figure 2:**
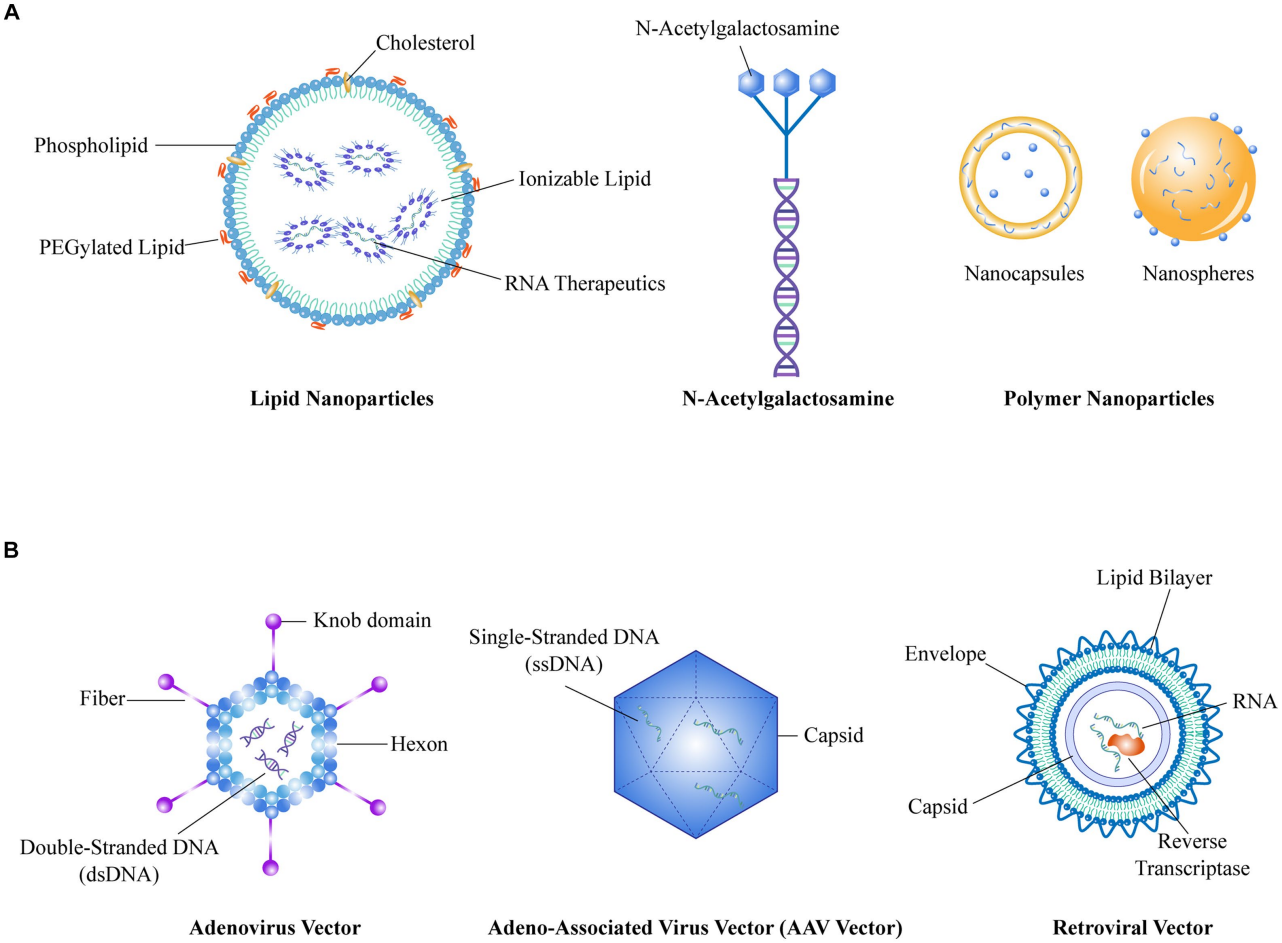
**(A)** Represents the schematic structure of a non-viral vector, **(B)** Represents the schematic structure of a viral vector.

Based on the biological attributes of biomimetic units, natural biomimetic nano-systems offer three primary advantages: extended circulation within the body, precise targeting capabilities, and reduced toxicity. Currently, natural biomimetic nano-systems in the field of drug delivery for RA treatment including endogenous albumin, extracellular vesicles, cell membranes, and genetically engineered membranes. These innovative systems have demonstrated effectiveness in delivering therapeutic drugs or NPs to the affected joints, thereby enhancing the overall therapeutic outcome (Figure 2).

### 4.1 Endogenous albumin

Human serum albumin (HSA), the primary component of serum proteins, serves as a versatile carrier for therapeutic and diagnostic drugs with inflammation-targeting properties. Given its naturally biocompatibility, biodegradability, ease of production, and cost-effectiveness, HSA stands as a promising multifunctional drug carrier (22–24). In recent years, several preclinical studies have reported albumin-based biomimetic nano-systems as drug delivery vehicles for RA treatment in animal models (25–27).

A previous study revealed the overexpression of secreted protein, acidic and rich in cysteine (SPARC) in the synovial fluid and synovium of both RA patients and collagen-induced arthritis (CIA) mice (28). Moreover, augmented metabolism of synovial cells was observed in inflamed joints compared to healthy tissues, necessitating increased utilization of albumin for nitrogen and energy. These metabolic alterations in inflamed joints were found to be associated with the occurrence of hypoalbuminemia in RA patients. Consequently, the specific aggregation of albumin at inflammation sites can be attributed to increased blood albumin consumption, augmented permeability, and upregulated expression of SPARC.

In 2019, Liu and colleagues successfully developed HSA-NPs with specific targeting abilities to enhance the safety and therapeutic effectiveness of methotrexate (MTX) in CIA mice (29). Subsequently, Lyu et al. (30) and Chen et al. (31) developed two types of mannose-modified MTX-loaded HSA-NPs (MTX-M-NPs) to further augment the targeting capabilities of albumin NPs to inflammatory sites in animals. These targeted nano-systems enables the precise delivery of therapeutic agents to neutrophils specifically at the inflamed joint site by binding to the mannose receptor on the surface of neutrophils. Consequently, the utilization of MTX-M-NPs holds significant potential in enhancing the anti-inflammatory capabilities of neutrophils at the joint site in patients with RA. The application of albumin NPs in RA treatment was summarized in Table 1.

**Table 1:** Application of albumin NPs in RA treatment.

NPs	Therapeutic advantages	Animal models
MTX-loaded albumin NPs (32)	MTX prodrug selectively binds to the cysteine-34 position of endogenous albumin and is efficiently cleaved by histone B and fibrinolytic enzymes overexpressed in RA and releases MTX.	CIA mice
Albumin-TRAIL coupling polymers (33)	Has significant targeted RA and long circulation	CIA mice
PAB-CLT (30)	PAB has significant SR-A targeting over BSA maleate (SR-A ligand)	AIA rat
HAS-PD/CU (34)	Accumulates in inflamed joints through the ELVIS effect and exhibits a slow release, synergistically delivering a high therapeutic effect at a low dose.	AIA rat
HAS-MTX (28)	Based on the high expression of SPARC in RA and the inherent high affinity of SPARC for albumin	CIA mice
MTX-M-NP_S_ (35)	Targeting of drugs to neutrophils by binding to mannose receptors	CIA rat

Albumin-based nano-systems offer advantages in enhancing targeted therapeutic efficacy at inflamed joint sites, as well as extending the half-life and improving the bioavailability of drugs. Fatty acids, such as palmitic acid, are commonly used to prolong the half-life of proteins or peptides. Accordingly, Gong et al. (36) synthesized palmitic acid (PA)-modified bovine serum albumin (BSA) NPs (PAB-NPs). Through *in vivo* pharmacokinetic experiments, it was demonstrated that PAB-NPs significantly prolonged the drug’s circulation time and improved its bioavailability compared to BSA-NPs. Targeting studies additionally revealed the prominent scavenger receptor-A (SRA) targeting properties of PAB-NPs, resulting in a remarkable 9.1-fold higher uptake of PAB-NPs by activated macrophages compared to BSA-NPs (36).

### 4.2 Extracellular vesicle

In recent years, significant advancements have been made in the field of RA treatment with regards to extracellular vesicles (EVs) (37, 38). These membrane-bound vesicles derived from various cells, play a crucial role as messengers in inter-cellular communication and the regulation of various pathophysiological conditions. EVs can be categorized into three primary subgroups based on their biological origin and size: exosomes (Exo, 30 to 200 nm), macrovesicles (MVs, 200 to 1,000 nm), and apoptotic vesicles (Avs, >1,000 nm) (39, 40). EVs can naturally be secreted by diverse cell types, such as macrophages and cancer cells, and are known for their non-cytotoxicity, non-immunogenicity, and excellent biocompatibility (41). In addition, the presence of consistent adhesion molecules on the EV surface facilitates preferential binding to host cells and helps them evade phagocytosis by endothelial reticulocytes (42, 43). EVs also play a crucial role in intercellular communication by transferring their cargo to various cells, thereby promoting cell transcription and proliferation (44). Therefore, the utilization of EVs as nano-systems for drug encapsulation can achieve effective drug delivery. Compared to cell-mediated nano-systems, EVs provide the advantage of reducing clearance by the mononuclear phagocyte system (MPS) and enhancing the accumulation of NPs in tissues (45).

Considering the involvement of macrophages in the inflammatory micro-environment of RA and their pro-inflammatory properties, researchers have explored the potential of using macrophage-derived EVs as drug carriers for RA treatment (46). Macrophages possess specific targeting properties due to the presence of surface membrane proteins, and EVs secreted by macrophages can inherit these targeting abilities from their host cells. Yan et al. (47) developed a biomimetic nanoparticle utilizing macrophage-derived Exo, wherein dexamethasone sodium phosphate (Dex) was encapsulated (Exo/Dex). The surface of these Exo was further modified with a folic acid (FA)-polyethylene glycol (PEG)-cholesterol (Chol) compound to create an active targeted drug delivery system known as FPC-Exo/Dex. Their results showed that the biomimetic drug delivery system exhibited an extended systemic circulation time for the drug, enhanced targeting efficiency at the site of inflammation, and offered enhanced protection against bone and cartilage damage in mice with CIA (47).

In another study, researchers utilized macrophage-derived micro vesicles (MMVs) encapsulated within NPs (MNPs) as a targeted approach for RA treatment (48). The proteomic profile of MMVs was analyzed using iTRAQ (isobaric tags for relative and absolute quantitation) labeling, providing insights into the relative and absolute protein levels. The presence of membrane proteins in MMVs that closely resemble those found on macrophage suggests that MMVs can exhibit similar biological activities to macrophage-targeted RA therapy. In addition, poly (lactic-co-glycolic acid; PLGA) NPs were encapsulated with MMVs, and the targeting efficacy of the MNPs system for inflammatory therapy was evaluated both *in vitro* and *in vivo*. Their results indicating that MNPs hold great promise as a biomimetic nano-delivery system for RA treatment (48). The application of EVs in RA treatment was summarized in Table 2.

**Table 2:** Application of EVs NPs in RA treatment.

NPs	Therapeutic advantages	Animal models
IL-10-treated DC-derived exosomes (49)	Ability to inhibit the onset of arthritis and reduce the severity of established arthritis	CIA mice
FPC-Exo/Dex (44)	Targeted activation of FRβ expressed by macrophages inhibits the secretion of pro-inflammatory cytokines and increases the expression of anti-inflammatory cytokines for better protection of bone and cartilage in CIA mice	CIA mice
MNP (45)	Enhancement of therapy by targeting ICAM-1 or p-selectin highly expressed by activated macrophages	CIA mice

### 4.3 Cell membrane

In recent years, there has been a surge of interest in the study of cell membrane-coated NPs owing to their remarkable biocompatibility, ability to retain cellular properties, and versatility in a wide range of therapeutic and imaging applications (50). Various immune cells have been identified to have crucial involvement in the progression of RA, and their cell membranes offer potential as nano-delivery systems with functionalities and targeting abilities. In addition, from a biological and immunological perspective, a novel interfacial attachment technique known as cell membrane capping technology has emerged as a promising approach to enhance the efficacy of synthetic nanocarriers (51). Apart from the extensively studied red blood cells (RBCs), various cell types such as platelets, white blood cells, cancer cells, stem cells, and even bacteria offer potential as sources for membrane materials, each possessing distinct properties and exerting diverse targeting characteristics (52, 53).

Upon coating with cell membranes, NPs not only acquire the physicochemical attributes of native cell membranes but also inherit distinctive biological functionalities arising from the presence of membrane-anchored proteins, antigens, and immune components (54, 55). The inherent biological properties and functions derived from these cell membrane-coated NPs, including immunosuppressive effects, prolonged circulation, and targeted recognition, underscore their significant potential in the field of biomedicine (56). Consequently, the development of a biomimetic nano-delivery system, emulating endogenous cells, holds promise for enabling molecular imaging and precise drug delivery to inflamed joint sites.

Erythrocytes, favored by researchers due to their remarkable circulatory longevity of up to 120 days, exhibit immense potential as carriers for drug delivery (57). Erythrocyte membrane-coated NPs have been proven to effectively extend the half-life in the systemic circulation, surpassing the performance of polyethylene glycol-coated nano-delivery systems (58). Li et al. reported a resveratrol-loaded PLGA NPs functionalized with erythrocyte membranes as a biomimetic delivery system significantly prolonged the circulation time of resveratrol in mice (32). During the last two decades, PEG has been the focus of studies due to its immunogenicity, which may trigger accelerated blood clearance (ABC) and hypersensitivity reactions to PEGylated NPs (59, 60). However, erythrocyte membrane-coated NPs are not affected by the “ABC” effect. The presence of CD47, a distinctive “do not eat me” protein expressed on the surface of RBCs, plays a crucial role in enabling the NPs to evade immune clearance by interacting with signal regulatory protein-α receptors (33). Furthermore, researchers have explored the intrinsic interaction between P-selectin expressed on platelets and inflamed tissues to develop platelet membrane NPs loaded with FK506 (tacrolimus), a potent immunosuppressant, for targeted treatment of RA at inflamed joint sites (34). These platelet membrane NPs exhibited prolonged drug circulation time in the bloodstream, enhanced accumulation at inflamed joint sites, and effectively mitigated joint swelling and inflammation.

In a previous study conducted by Dehaini, a novel bio-coating was developed using fused cell membranes of RBCs and platelets, resulting in RBC-platelet membrane NPs (49). The fused membrane combines the functionalities of erythrocytes and platelets, and experimental findings indicated that this carrier possesses properties from both cell sources. This innovative approach paves the way for the development of biomimetic nano-delivery systems with diverse functionalities, tailored to overcome existing limitations of nanoparticle-based therapeutic and imaging platforms (49).

Neutrophils have been observed to accumulate at inflammation sites in RA, and play an important role in reducing inflammation and repairing tissue damage (61). Zhang et al. developed neutrophil membrane-encapsulated NPs by fusing neutrophil membranes onto polymer cores (62). The resulting nano-delivery system retained the relevant membrane functions and antigenic properties of the host cells, making it an excellent candidate for targeted delivery to neutrophils. The NPs exhibited the ability to inhibit the secretion of pro-inflammatory cytokines, attenuate synovial inflammation, and provide protection against bone and cartilage damage in both CIA models and human transgenic arthritis mouse models (62). The application of cell membrane nano-system in RA treatment was summarized in Table 3.

**Table 3:** Application of cell membrane NPs in RA treatment.

NPs	Therapeutic advantages	Animal models
Neutrophil membrane-encapsulated PLGA NPs (57)	LFA-1 on neutrophil membranes binds to ICAM-1 and enhances targeting, a function-driven, broad-spectrum and disease-associated blocker that inhibits inflammatory cascades in disease processes	CIA mice/human TNF-α transgenic mice
Platelet membrane-encapsulated PLGA NPs (62)	Targeting disease sites by P-selectin and GVPI recognition	CIA mice
TU-NPs (63)	Neutralizes cytokines, inhibits synovial inflammation and provides strong cartilage protection to prevent joint-damaging substances from penetrating deep into inflamed tissues.	CIA mice

### 4.4 Genetically engineered membrane

The advancements in naturally biomimetic nano-delivery systems have transformed cell membrane-coated NPs into a viable and practical therapeutic platform (64). In order to enhance the targeted delivery capabilities of nano-systems, it is possible to modify them with specific ligands that target inflammatory tissues or cells. However, certain chemical or physical modifications may potentially disturb the structure or functionality of proteins present in cell membranes. Nevertheless, genetic engineering provides a means for the specific expression of targeted ligands onto cell membranes, without disrupting the existing membrane proteins. Taking advantage of this, researchers utilized genetic engineering techniques to generate cell membranes expressing tumor necrosis factor-related apoptosis-inducing ligand (TRAIL) from human umbilical vein endothelial cells (65). Subsequently, the fusion of the TRAIL-anchored membrane with hydroxychloroquine-loaded PLGA-NPs enables targeted delivery to activated M1 macrophages at the inflammation site, with the goal of therapeutically suppressing the secretion of pro-inflammatory. Following intravenous injection, the bionanoparticles were observed to accumulate and persist in the inflamed joints, leading to a favorable anti-inflammatory therapeutic outcome (65).

### 4.5 Bacteria

Remarkable progress has been made in the research of utilizing bacteria as a natural biomimetic nano-system for drug delivery in RA treatment. Studies primarily focus on harnessing the unique attributes of bacteria, such as their inherent targeting capabilities, programmability, and biocompatibility, to develop novel therapeutic approaches (66, 67). Through genetic engineering techniques, researchers have successfully engineered bacteria to target inflammatory sites in RA and subsequently release anti-inflammatory drugs or bioactive molecules, including anti-inflammatory proteins, immune modulators, siRNA, and miRNA, upon reaching the designated location. A preclinical study by Fan et al. developed an orally administered light-activated bacterial system that can specifically release TNF-α at inflammatory sites for tumor treatments, demonstrating the potential of utilizing bacteria for targeted therapy (68). Tao et al. presented a novel approach for the highly effective and dual-selective ablation of hypoxic tumors using engineered bacteria sensitized with near-infrared nanoantenna (69). These breakthrough methods hold promise as a new conceptual framework for potential applications in the treatment of RA.

In addition, outer membrane vesicles (OMVs) play crucial roles in various bacterial physiological activities and pathogenicity. Leveraging the physiological characteristics of OMVs, delivery of therapeutic substances such as siRNA, miRNA, and proteins to tissues has been achieved (70, 71). Effective liposomal nanocarriers designed through biotechnology methods have enhanced targeting drug delivery and immunogenicity through homologous and heterologous antigen modification (70). The lipid bilayer topology of liposomes allows for encapsulation of amphiphilic therapeutic drugs, which not only increases their stability and reduces side effects but also prolongs their half-life. Liposome encapsulation of adjuvant chemotherapy drugs for the treatment of colorectal cancer is considered a promising targeted drug delivery system. OMVs can serve as natural or engineered carriers of cell-protective factors or cytotoxins, making them a novel therapeutic tool applicable from regenerative medicine to targeted cancer therapy (72). To promote the release of therapeutic drugs under specific conditions, further research based on OMVs is required to engineer liposomal nano-carriers, thereby improving targeting specificity and increasing the uptake of therapeutic drugs.

While study on the use of bacteria-based nano-systems for drug delivery in the treatment of RA is currently limited, it is important to acknowledge the several key challenges that must be addressed before undertaking further studies. These challenges include ensuring the overall safety of bacterial carriers, improving delivery efficiency and precision, and achieving precise control over drug release. In summary, bacteria-based nano-systems hold great promise for the development of innovative treatments in the field of RA.

## 5 Nucleic acid delivery system

Nucleic acids are biocompatible materials with unique properties and structures. Small molecule nucleic acids such as sgRNA, siRNA, and shRNA can be specifically employed to silence target proteins, making them valuable tools for targeted delivery in nanomaterial applications. RNA is a versatile biomolecule present in biological cells as well as certain viruses and viroids. In a broader sense, RNA can be categorized into two types: coding RNA and non-coding RNA. Coding RNA refers to mRNA, which can be translated into proteins. On the other hand, non-coding RNA encompasses various types, including rRNA, tRNA, siRNA, miRNA, and antisense oligonucleotides (ASO), among others (73). In the field of drug research, small molecule drugs and protein-based therapeutics hold a dominant position, as these molecules function by acting on downstream target proteins of disease-causing genes. However, there is a lack of targeted drugs for many disease-related proteins, necessitating the exploration of more precise and effective therapeutic strategies.

**Figure 3:**
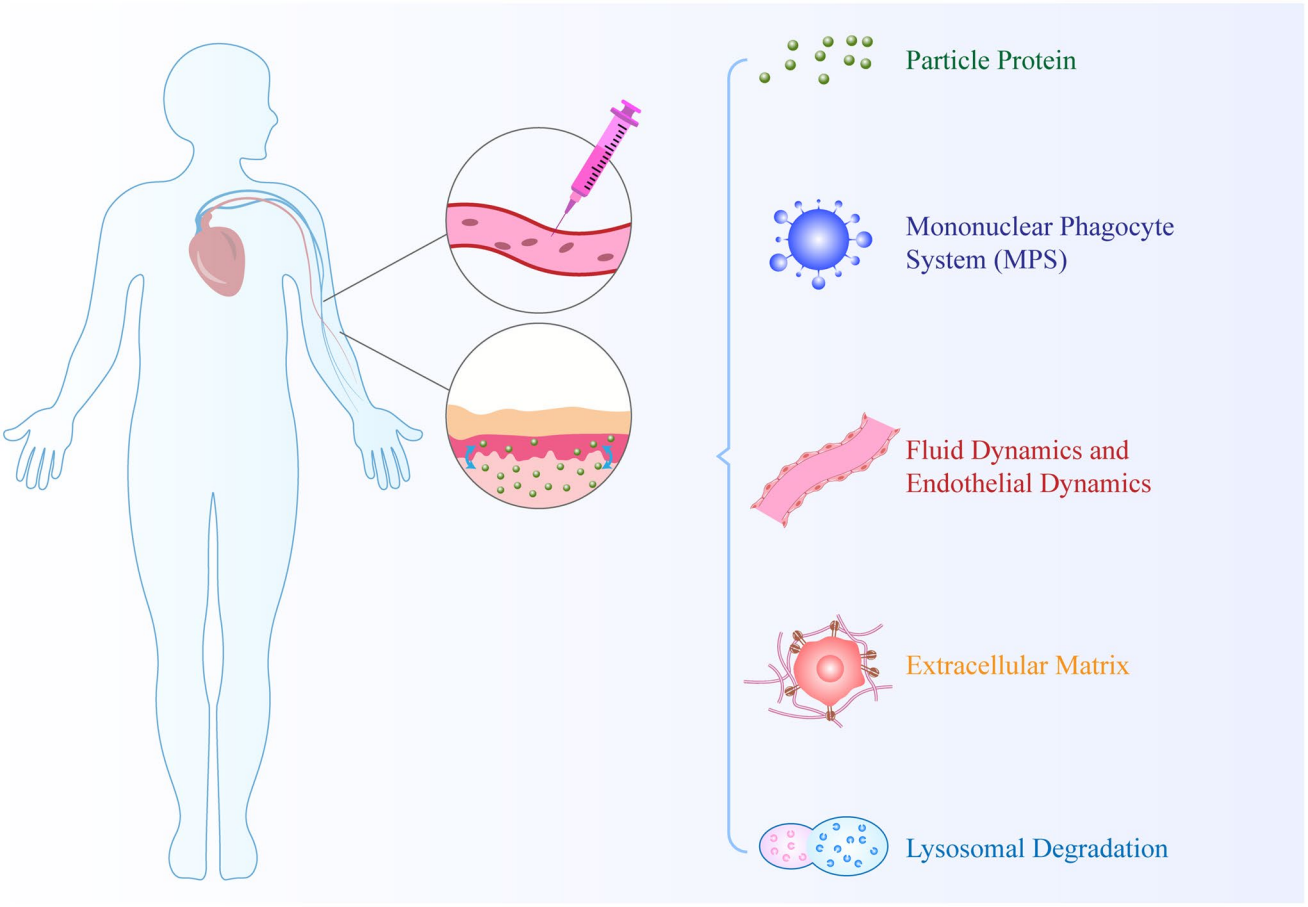
Biological transport barriers of nanodrug.

The advent of RNA interference (RNAi) technology has revolutionized the ability to manipulate molecular processes with unprecedented precision (74). Compared to DNA, RNA is less stable and therefore requires more demanding delivery vehicles. Based on the composition of delivery system, RNA delivery vehicles can be broadly categorized into non-viral vectors and viral vectors (Figure 3; Table 4).

**Table 4:** Classification and characteristics of RNA Carriers.

Classification	NPs	Structure	Advantages	Disadvantages
Non-viral vectors	LNPs	Lipid vesicles with uniform lipid core	Uniform packaging, efficient intracellular and extracellular nucleic acid expression, high safety profile	Limited tissue targeting specificity
	GalNAc	Trivalent sugar compounds	Suitable for subcutaneous administration, scalable in manufacturing, with a high safety profile.	Limited to targeting hepatocytes
	PNPs	Product formed by polymerization of a monomer	Controlled drug release rate, biodegradable and biocompatible, with target specificity	Encapsulation High of toxicity, hydro residualphil organicic sol drugsvents, challenges insufficient in encaps largeulation of-scale hydro productionphilic, drugs and, issues challenges in with large storage-scale production and, and sterile issues withization storage
	INPs	Synthesis of Inorganic Particles and Biodegradable Polycation	Hydrophilic, biocompatible, and highly stable	Lack of clinical trial data, challenging clinical translation
Viral vectors	AdV	Large molecule double stranded uncoated icosahedral DNA virus	High thermal stability and strong ability to induce innate immunity, with only transient expression	immunogenicity, limited vector capacity, and restricted intracellular replication
	AAV	Icosahedral DNA deficient virus with non-enveloped single stranded linear structure	High safety profile, low immunogenicity, broad spectrum of infection, prolonged expression of exogenous genes *in vivo*	Limited size of the target gene fragment, delayed expression post host cell infection
	RV	Encapsulated spherical RNA virus	Broad infection spectrum, high specificity, high integration efficiency, capable of stable expression of the target gene	Presence of insertional mutations, potential risk of oncogene activation, with limited vector capacity
	LV	Encapsulated spherical RNA virus	Wider infection spectrum, high specificity, high integration efficiency, capable of stable expression of the target gene, with increased vector capacity	Presence of insertional mutations and potential risk of oncogene activation
Viral-like vector	VLP	Highly structured protein particles	High specificity and biological activity, high safety profile, high delivery efficiency	High immunogenicity

### 5.1 Non-viral vectors

Non-viral vectors primarily include lipid NPs (LNPs), N-acetylgalactosamine (GalNAc), polymer NPs (PNPs), and inorganic NPs (INPs) (75, 76). With their flexible size, shape, structure, low toxicity and accessible surface modification, non-viral vectors show great promise for application in RNA delivery. Among them, LNPs have found widespread application and are considered the optimal carriers for mRNA vaccines (75). However, their utilization is mainly limited to liver tissue targeting (77), and there is still a need for develop targeting capabilities toward extra-liver tissues. LNPs have been widely acknowledged as a promising delivery approach in the treatment of RA, as summarized in a previous review article (78).

### 5.2 Viral vectors

Viral vectors, consisting of adenovirus vectors (AdV), adeno-associated viral vectors (AAV), retroviral vectors (RV), and lentiviral vectors (LV), are prominent vehicles for RNA delivery (79). These viral vectors possess advantages such as broad and targeted transduction capabilities, high delivery efficiency, and prolonged expression profiles. Among them, AAV vectors offer high delivery efficiency and have already been applied in clinical gene therapy for both *in vivo* and *ex vivo* applications, making them a relatively mature delivery technology (80). However, AAV vectors have limitations in terms of the size of the target gene fragment they can accommodate and the delayed onset of gene expression after infection of host cells, highlighting the need for ongoing optimization.

### 5.3 Viral-like vectors

Virus-like particle (VLP) vectors stand as an innovative gene therapy platform that has been developed in recent years, providing a novel approach for RNA delivery (81). VLPs are highly structured protein particles that self-assemble from one or multiple viral structural proteins. They resemble the morphology and structure of their corresponding natural viruses, thereby exhibiting strong immunogenicity, specificity, and biological activity (82, 83). Importantly, VLPs do not contain viral nucleic acids, rendering them incapable of replication and thus offering enhanced safety (84). The VLP delivery system utilizes the recognition principle between mRNA stem-loop structures and phage coat proteins. Through the utilization of viral engineering techniques, the advantages of both viruses and mRNA are synergistically combined, leading to the development of a novel delivery technology known as VLP-mRNA. This emerging platform has garnered significant attention as the next frontier in RNA delivery carriers.

## 6 Limitations and prospects

While nano-system for drug delivery holds immense potential in overcoming challenges in disease treatment and diagnostics by leveraging the properties of nanomaterials, some inherent limitations should be considered, particularly concerning the potential for cellular toxicity at the cellular level (85). Upon cell exposure, nanomaterials can cause varying degrees of cell damage, resulting in the generation of reactive species such as reactive nitrogen and oxygen species (86). Therefore, in addition to assessing the therapeutic properties of the drugs themselves, it is crucial to evaluate the toxicological impact of nanomaterials to ensure their safety. However, this process is costly and may block nanomedicines promoting to clinical trials.

**Figure 4:**
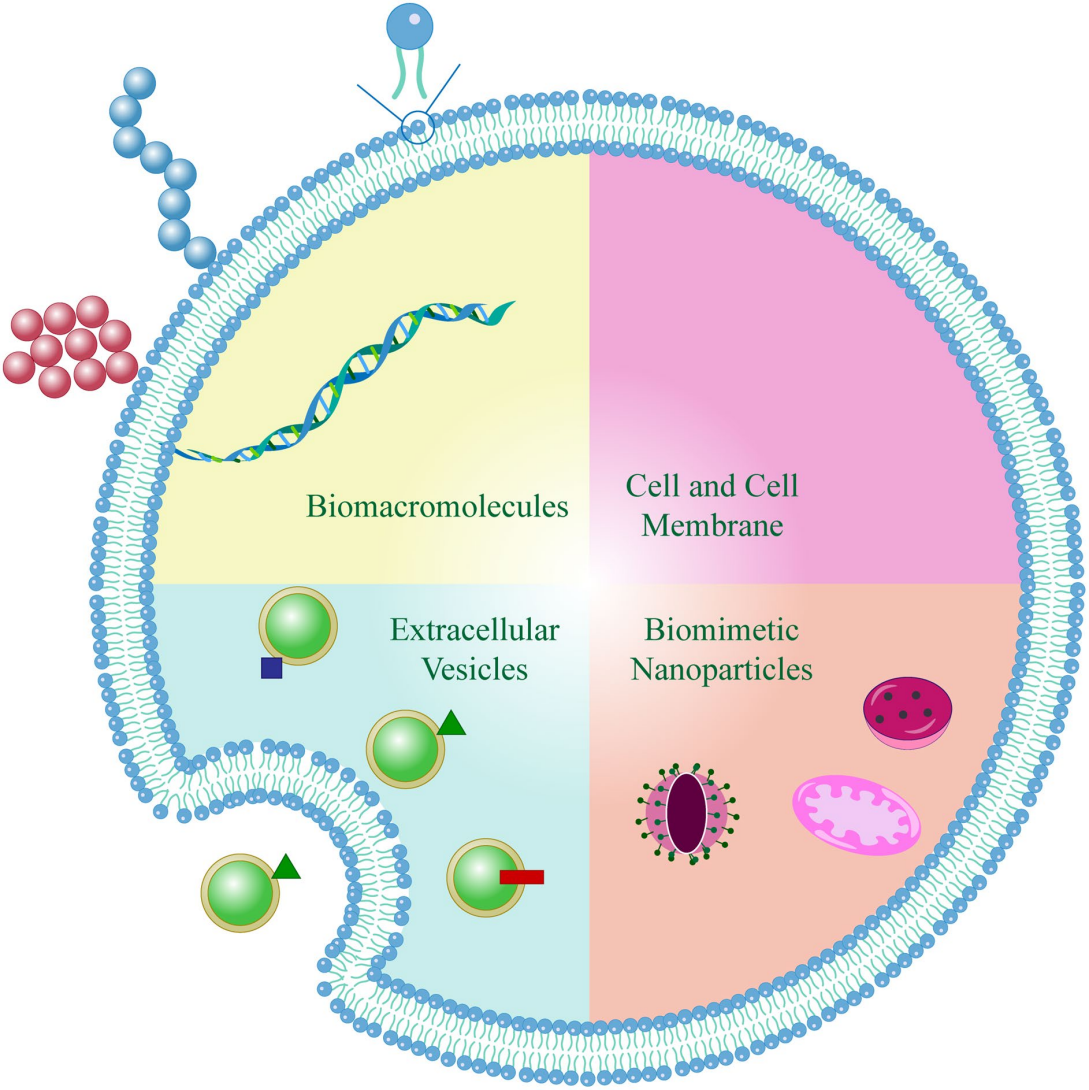
Basic unit of biomimetic nanoparticle design.

Nanocarrier-based drug delivery systems encounter several biological barriers in drug transport. These transport processes occur within different compartments, such as within the cytoplasm and between compartments. As shown in Figure 4., biological barriers, including cell membranes, nuclear membranes, and endosomal membranes, significantly interfere with drug delivery (87). Firstly, upon contact with biological fluids, NPs accumulate molecules on their surface and form a protein corona. The dynamic multi-layer protein structure of the protein corona provides NPs with specific identities, which can influence their physicochemical properties and subsequent biological interactions and distribution. Secondly, the activation of resident macrophages in the reticulate endothelial system (RES) aids in the clearance of old blood cells and substances carried in the blood circulation to RES organs. A key limitation of nanomedicines is their rapid phagocytosis and clearance based on the RES, resulting in a decrease in the bioavailability of nanodrugs. Thirdly, NPs face complex fluid dynamics when passing through curved and bifurcated regions of healthy or diseased blood vessels (88). Fourthly, the extracellular matrix provides tissues with structural integrity, characterized by high collagen content, rigidity, and tensile strength. It serves as a major natural physical barrier that hinders the delivery of nanodrugs. Finally, once NPs extravasate from blood vessels to the site of infection, they can bind to cell membranes, leading to internalization. This highlights the challenges faced by nanotechnology in drug delivery. However, the development of targeted nanomedicines that can directly deliver drugs to the inflamed site by targeting molecular constituents involved in RA pathophysiology or immune cells can potentially overcome these barriers.

## 7 Conclusion

The clinical treatment RA poses several challenges, making the development of endogenous substances as drug delivery systems necessary. Endogenous albumin, extracellular vesicles, cell membranes, nucleic acids, and bacteria have been chosen as biomimetic nano-delivery systems. These systems are preferred not only for their non-immunogenic and low toxicity properties but also for their capability to effectively evade immune system clearance and prolong drug circulation in the body. However, the use of bacteria as biomimetic nano-delivery systems is still in the exploratory stage, and several challenges need to be addressed, such as ensuring comprehensive safety of bacteria carriers, enhancing delivery efficiency and accuracy, and achieving precise control over drug release. Delivery carriers have improved the stability and target specificity of RNA formulations, thereby facilitating the clinical application of RNA-based therapeutics. Non-viral carriers, characterized by their low toxicity, high safety, large payload capacity, and design flexibility, offer several advantages. Among them, LNPs have wide-ranging applications and are considered the optimal carriers for mRNA vaccines. Viral carriers, on the other hand, exhibit broad-spectrum and strong targeting capabilities, high delivery efficiency, and sustained gene expression. Among viral carriers, AAV vectors have demonstrated high delivery efficiency and represent a relatively mature delivery technology. However, AAV vectors have limitations, such as restricted payload capacity for the target gene fragment and delayed expression after host cell infection, necessitating further optimization. VLP delivery strategies show great potential for efficient intracellular delivery of mRNA, offering broad applicability. In conclusion, further exploration is required to advance the clinical trials and applications of potential drug delivery systems in the field of RA, building upon existing biomimetic nano-delivery systems.

## Author contributions

JL: Writing – review & editing. WL: Writing – review & editing. LZ: Writing – original draft, Writing – review & editing.
